# Patient experience of health and care when undergoing colorectal surgery within the ERAS program

**DOI:** 10.1186/s13741-020-00144-6

**Published:** 2020-05-20

**Authors:** Berith Wennström, Anna Johansson, Sabina Kalabic, Anna-Lena E-son Loft, Stefan Skullman, Ingrid Bergh

**Affiliations:** 1 Department of Anaesthesia, Skaraborg Hospital, Skövde, Sweden; 2 School of Health Sciences, University of Skövde, Skövde, Sweden; 3 Department of Surgery, Skaraborg Hospital, Skövde, Sweden; 4 Research and Development Center, Skaraborg Hospital, Skövde, Sweden

**Keywords:** Colorectal surgery, ERAS, Patient experiences, Telephone follow-up, State of health

## Abstract

**Background:**

Several studies show that the enhanced recovery after surgery (ERAS) program reduces complications postoperatively and leads to faster recovery and shorter hospital stays. However, little is known about patients’ self-reported health in an enhanced recovery context. The aim of this study was firstly to describe patient experiences of health within the concept of ERAS after colorectal (CR) surgery during a hospital stay and within 2 weeks of discharge. Secondly, to explore whether the ASA classification/co-morbidity, sex, and surgical method affect the patient’s experience of health.

**Methods:**

Data were collected through the ERAS-HEALTH questionnaire, including two open-ended questions, and through telephone interviews postoperatively. Qualitative and quantitative analysis was used. Patients undergoing CR surgery (*n* = 80) were included from October 2016 to June 2018.

**Results:**

The patients had mainly positive experiences of their hospital stay as well as most of them felt comfortable coming home. However, experienced state of health is affected by factors like surgical method and co-morbidity. Improvements were desired concerning information, food/food intake, pain management, and environment. At home, the patients experienced a lack of information about food/food intake and ostomy care. Decreased appetite and difficulties with micturition were also described. The most troublesome symptom was postoperative fatigue (POF). Analysis of the ERAS-HEALTH questionnaire showed that patients with higher co-morbidity and those who underwent open surgery have a significantly worse experience of their health compared with patients who underwent laparoscopy. However, it seems that the surgical method affects postoperative health to a greater extent than co-morbidity.

**Conclusions:**

The patients reported many positive aspects and challenges when being cared for within the ERAS program. However, several improvements are needed to satisfy patient wishes regarding their care both in hospital and at home. Laparoscopic surgery affects patient state of health positively in several respects compared with open surgery.

## Introduction

Traditionally, recovery after colorectal (CR) surgery has been associated with bed rest and long hospital stays, sometimes up to 14 days. Postoperatively, eating and drinking were almost forbidden until return of bowel function was achieved, which increased the risk of malnutrition (Fearon and Luff, 2003). In addition, recovery at home was slow and morbidity as well as mortality was high during the first month postoperatively (Slater, 2010). Today, health professionals advocate multimodal prehabilitation programs to enhance patients’ functional capacity and to reduce postoperative complications. This intervention may result in increased survival and improved health-related quality of life (HRQoL) (van Rooijen et al. 2019).

Enhanced recovery after surgery (ERAS) is a multimodal and multidisciplinary program that addresses evidenced best practice where the focus is to strengthen the patient’s physical and mental health and well-being perioperatively to major surgery. ERAS strives to minimize complications such as pain, stress, nausea, thrombosis, atelectasis, pneumonia, dysfunctional bowel function, and organ failure, which, in turn, contributes to faster recovery and earlier discharge. Being relatively free from pain and being able to handle it also reduces postoperative nausea and vomiting (PONV) and stress. All in all, this does not only reduce the patient’s suffering but is also cost-effective (Kehlet, 2008; Lau and Chamberlain, 2017; Ljungqvist et al. 2017).

The ERAS program encompasses the whole care chain perioperatively and assumes cross-professional (nurses, physicians, nurse assistants, dieticians/physiotherapists/occupational therapists), and interdisciplinary (surgeons/anesthesiologists) teamwork, according to the same guidelines and goals, so that the patient can recover as quickly as possible before discharge. This also requires that patients—as well as their relatives—are well-informed, prepared, and have knowledge of the planned care since careful information about the disease and treatment increases the patients’ ability to be optimally prepared to participate actively in their self-care and recovery, which, in turn, creates safety and confidence (Larsson et al. 2011).

The request for rapid recovery and short-term care leads to greater demands on both health professionals and patients. The ERAS preparation process therefore starts early so that the patient already in connection with the preoperative visit at the outpatient clinic is prepared both physically and mentally before surgery. This information is repeated on the day of admission including strict guidelines on how the care should continue.

ERAS is increasingly used, both nationally and internationally (Gustafsson et al. 2019). The program shows significant improvement in recovery for patients after surgery (Kehlet, 2008; Lau and Chamberlain, 2017; Ljungqvist et al. 2017). The principles for faster and safer recovery after surgery mediated by ERAS appear more and more as the golden standard adopted internationally to coordinate multi-professional and multidisciplinary collaboration for optimized care before, during, and after surgery.

Studies within ERAS have shown that it is possible to reduce length of stay at the hospital by up to 30% and complications by up to 48% (Spanjersberg et al. 2011; Varadhan et al. 2010). Early and common complications such as pain and postoperative fatigue (POF) are significantly reduced, which contributes to the patient’s well-being postoperatively (Anderson et al. 2003; Greco et al. 2014). However, a longitudinal study shows that POF, depression, and muscle weakness are common up to 1–6 months postoperatively (Jakobsson et al. 2014).

Few studies have investigated the patient’s experience of health and being cared for within the ERAS program both during the hospital stay and after discharge. Examining and evaluating ERAS care processes from a patient perspective is important as this can highlight both strengths and weaknesses of the ERAS approach in surgical care.

The aim of the study is two-fold. Firstly, to describe patient experiences of health within the concept of ERAS after CR surgery during hospital stay and within 2 weeks of discharge. Secondly, to explore whether ASA classification (co-morbidity), sex, and surgical method affect the patient’s experience of health postoperatively.

## Methods

### Participants and study design

This is a single institution study. However, the ERAS concept is widely implemented in several hospitals in Scandinavia and used for patients undergoing major elective surgery.

**Fig. 1:**
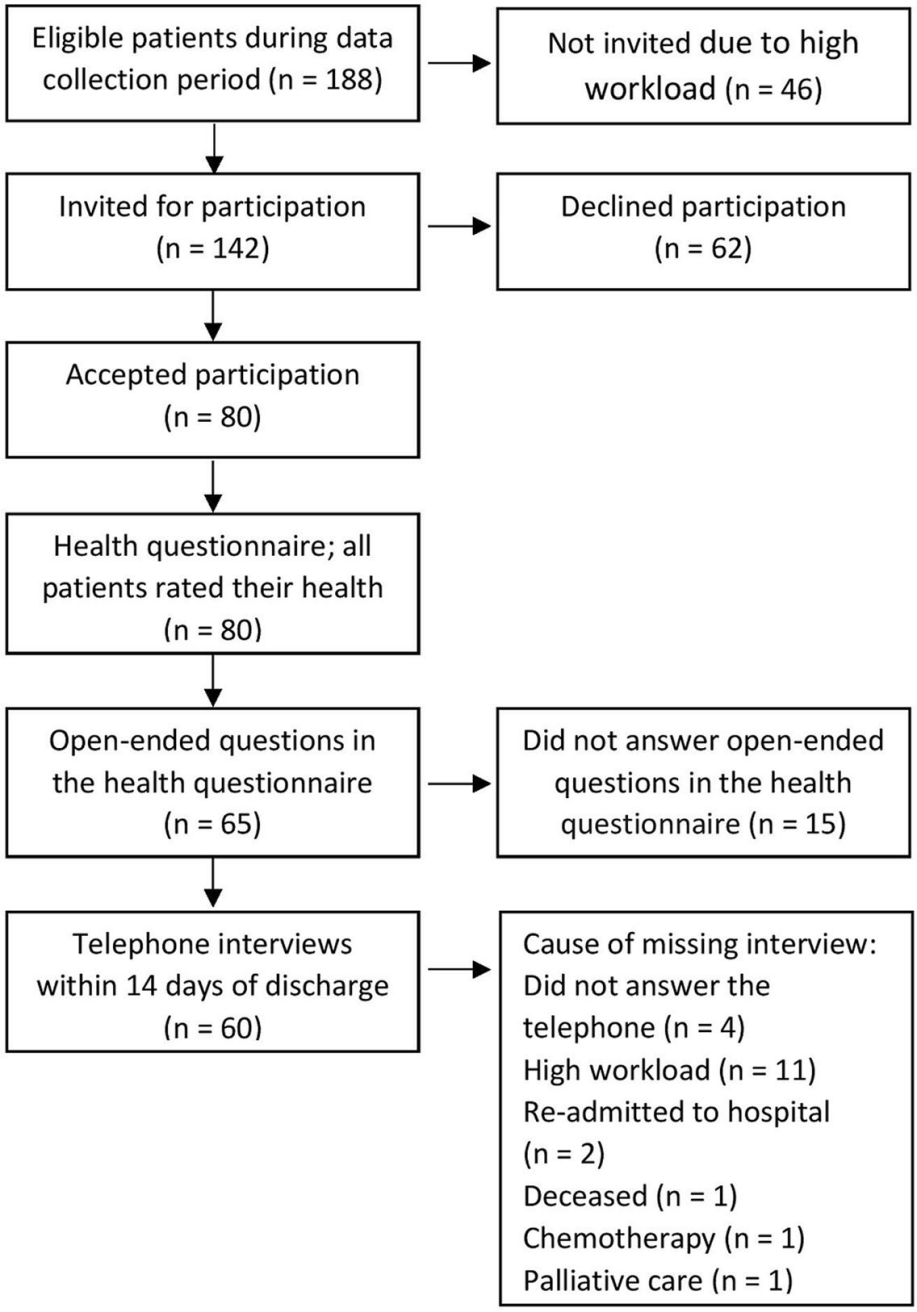
Data collection process

Patients, ASA class I-III, planned to undergo elective CR surgery with an intention to follow the ERAS program, were included from October 2016 to June 2018. The criteria for inclusion were the ability to understand and complete questionnaires in Swedish and the ability to take part in the planned telephone interview postoperatively. During this period, 188 patients were eligible for inclusion. Due to a high workload, 46 of these patients were not invited. In total, 142 patients were invited to participate in the study in connection with the preoperative admission appointment in the ward 1-2 days before surgery, where 80 of them chose to participate. Data were collected through the ERAS-HEALTH questionnaire, including two open-ended questions in connection with discharge and through telephone interviews within 2 weeks at home. The two open questions in the questionnaire were formulated to capture the patients’ descriptions (in their own words) of how they experienced their state of health and the care given to them. The design and the participant flow throughout the study are presented in Fig. 1. Patient demographics are presented in Table 1.

**Table 1:** Demography of the patients included in the study

Variables	Laparoscopic surgery ***n*** = 42		Open surgery ***n*** = 38		***p*** value	
	Men ***n*** = 19	Women ***n*** = 23	Men ***n*** = 24	Women ***n*** = 14	Laparoscopic surgery	Open surgery
Age, mean (SD)	70 (8)	69 (10)	70 (11)	69 (11)	0.693	0.732
Hospital stay, median (min-max)	4 (1–20)	3 (1–12)	8 (2–25)	5.5 (2–15)	0.361	0.139
**ASA*n*(%)**						
1	5 (26)	9 (39)	5 (21)	4 (29)		
2	12 (63)	13 (57)	14 (58)	6 (43)		
3	2 (11)	1 (4)	5 (21)	4 (29)		
**Diagnosis*n*(%)**						
Rectal cancer	11 (58)	13 (57)	12 (50)	7 (50)		
Colon cancer	8 (42)	7 (30)	10 (42)	6 (43)		
Morbus Chron	0	1 (4)	0	1 (7)		
Rectal prolapse	0	2 (9)	0	0		
Rectal injury	0	0	1 (4)	0		
Colovesical fistula	0	0	1 (4)	0		

### Procedure

To achieve variation in perspectives regarding patients’ experience of health, both quantitative and qualitative methods were used to collect and analyze the data. Data collection was conducted in three different steps:

- Step 1. In connection with discharge from the hospital, the patients were asked to complete the ERAS-HEALTH questionnaire. The ERAS-HEALTH comprises ten different symptoms affecting postoperative state of health (see Table 2) and its design is similar to that of the Edmonton symptom assessment scale (ESAS) (Bruera et al. 1991). The ESAS questionnaire has been translated into Swedish and used among cancer patient in palliative care (Astradsson et al. 2001).
- Step 2. The patients also responded in writing, in their own words, to two open-ended questions attached to the ERAS-HEALTH: (a) *Was there anything you missed or that could have done differently?* (b) *Describe in your own words your experience of well-being during your hospital stay*.
- Step 3. A telephone interview was conducted within 2 weeks of discharge by two experienced contact nurses who were used to interviewing. The interview question used is in accordance with Table 4. The patients also rated their current pain related to the surgical wound on a numerical rating scale (NRS), where 1 = no pain and 10 = worst possible pain. Notes were taken during the interviews.

**Table 2:** Association between ASA classification, surgical method, sex, and state of health variables using a multivariate model (ordinal regression). Univariate test results (Mann-Whitney test) are also presented

	Univariate test^**a**^	Multivariate model^**b**^	
State of Health	***p*** value	Estimate	***p*** value
**Pain**			
ASA classification	**0.030**	0.457	0.101
Surgical method	0.379	0.087	0.724
Sex	0.734	0.166	0.504
**Worry/anxiety**			
ASA classification	0.613	− 0.184	0.552
Surgical method	0.846	− 0.126	0.662
Sex	0.865	− 0.133	0.647
**Fatigue**			
ASA classification	**0.008**	0.635	**0.032**
Surgical method	**0.005**	0.732	**0.005**
Sex	0.492	0.375	0.143
**Nausea**			
ASA classification	0.353	0.398	0.332
Surgical method	**0.025**	0.765	**0.032**
Sex	0.328	0.593	0.092
**Depression**			
ASA classification	0.519	0.284	0.420
Surgical method	0.306	0.375	0.227
Sex	0.367	0.422	0.174
**Drowsiness**			
ASA classification	**0.009**	0.799	**0.009**
Surgical method	**0.008**	0.592	**0.023**
Sex	0.443	0.396	0.128
**Appetite**			
ASA classification	0.872	− 0.054	0.842
Surgical method	**0.026**	0.376	0.128
Sex	0.651	− 0.094	0.705
**Breathlessness**			
ASA classification	0.931	− 0.100	0.777
Surgical method	0.229	0.148	0.646
Sex	0.084	− 0.597	0.078
**Well-being**			
ASA classification	0.978	− 0.142	0.622
Surgical method	**0.004**	0.659	**0.014**
Sex	0.557	− 0.103	0.700
**Quality of Health**			
ASA classification	0.567	− 0.363	0.206
Surgical method	**0.001**	0.832	**0.002**
Sex	0.728	− 0.070	0.794

^a^Mann-Whitney *U* test

^b^Ordinal regression including three variables

Clinical and demographic data on sex, age, the American Society of Anaesthesiologists (ASA) physical status classification, diagnosis, and method of surgery were retrieved from the patient’s medical record (Table 1).

### Data analyses

#### Qualitative analysis

Qualitative content analysis was used to explore the patients’ answers to the two open-ended questions. Characteristic of this type of analysis is reading the text and focusing on the latent content a stepwise process of categorization based on the expression of thoughts, feelings, and actions described throughout the text. The analysis was guided by the descriptions of (Graneheim and Lundman, 2004) and was performed in three steps: (1) The qualitative data were condensed and grouped into codes by multiple interpretations and readings of the texts; (2) the codes were grouped into subthemes, and (3) the subthemes were abstracted into main themes. Patients could belong to more than one main theme based on their open-ended answer to the two questions. An overview of the main themes and subthemes is presented in Table 3.

**Table 3:** Overview of main themes and subthemes regarding patient experiences of health and care in connection with discharge from the hospital

Main themes	Subthemes
The meaning of information and communication	Feeling safe
	Being in control
	Being prepared
The meaning of health professionals’ competence, treatment and commitment	Feeling well cared for
	Feeling trust
	Feeling of participation
	Lacking confirmation
The meaning of experienced ill-being	Experiencing physical ill-being
	Experiencing psychological ill-being
The meaning of the healthcare environment and pleasant dining experiences	Feeling comfortable and content
	Experiencing reduced control of own integrity

#### Quantitative analysis

The notes from the telephone interview were analyzed using quantitative content analysis meaning that the patients’ answers were counted, summarized, and presented as frequencies (Krippendorff, 2004; Sandelowski, 2004).

The Mann-Whitney *U* test was used to examine differences in rated postoperative health based on sex, ASA classification, and surgical method (laparoscopic or open surgery) (Suppl. tables; A, B, C). An ordinal regression model (McCullagh, 1980) was used to assess whether the independent variables of sex, ASA classification, or surgical method were associated with the patient’s postoperative state of health (dependent variable) (Table 2). Descriptive statistics such as mean and standard deviations were also used. The statistical analysis was performed using the Statistical Package for the Social Sciences (SPSS version 22.0). *P* values < 0.05 two-tailed were considered statistically significant.

## Results

### Patient experience of health and care in connection with discharge

The responses to the semi-structured questions resulted in four main themes and 11 subthemes. Main themes, such as “The meaning of information and communication,” “The meaning of health professionals’ competence treatment and commitment,” “The meaning of experienced ill-being,” and “The meaning of the healthcare environment and pleasant dining experiences”, describe—together with the subthemes—the experienced state of health, as well as the importance of the different healthcare staff approaches within this context (Table 3).

#### The meaning of information and communication

Patients who felt that they had received adequate information could plan their rehabilitation period better, which provided safety, control, and a feeling of being prepared.

*“Very good and clear information at admission, which prepared me well for what was going to happen before and after surgery. This helped me to focus forward. I felt safe and cared for after the operation. Considerably less pain than I expected.” (Patient 23)*

In some cases the information was perceived as deficient and sometimes even incorrect.

*“The first meal consisted of ropy meat, pineapple pie and long pieces of vegetables (peppers) with the skin left on, which is exactly what you should avoid when you have just had surgery and there’s a risk of slowed-down bowel movements.” (Patient 8)*

#### The meaning of the health professionals’ competence, treatment, and commitment

Commitment on the part of the staff was seen as an important aspect in order for the patient to feel perceived as a unique individual, to be acknowledged. This gave rise to feelings of kindness, gratitude, and thoughtfulness as well as an experience of participation.

*“I’m touched by the commitment I have received from everyone, regardless of their profession. Impressed with how the staff put patients into focus responsiveness helpfulness patience for all the questions one has. The staff is kind, considerate and cares for the patient.” (Patient 52)*

Poor treatment was experienced when the staff ignored the patient and his/her symptoms: an absence of confirmation and disappointment at not being taken seriously.

*“It was ridiculous that no one bothered with the diary that I had filled in. The staff said that they had filled in their own. I think it would have been really interesting to see if they matched. For example, the staff didn’t have a clue about how much I’d sat in the chair or walked down the corridor, or how often I felt sick or in pain, etc. They only asked once a day and then wanted to know how I felt at that precise moment.” (Patient 8)*

#### The meaning of experienced ill-being

Ill-being was experienced both physically and mentally when the patient described loss of appetite, powerlessness, pain, nausea, and anxiety concerning the whole situation.

*“The negative element regarding quality of life and well-being has to do with the fact that I was affected by this disease. My cancer became the culmination of depression and loneliness.” (Patient 66)*

*“Before the operation I was really anxious. Now I feel some fatigue and drowsiness and nausea every now and again. No appetite at all due to the medication.” (Patient 9)*

There was also dissatisfaction with the pain management strategies and an experience of long waiting times for pain relief treatment.

*“The doctor said during the round that they would provide pain relief but it doesn’t seem to work properly … different treatment by different nurses. In some cases, the time between the need for pain relief and actually getting it felt too long.” (Patient 63)*

#### The meaning of the healthcare environment and pleasant dining experiences

The patients’ experience did not only include the physical environment but also the mental and social environment, including the pros and cons of single or twin rooms. The benefit of being cared for in a single room included having one’s own toilet and shower and not having to worry about disturbing someone else. It was easier to receive visitors/relatives and the patient’s integrity was enhanced. Twin rooms were appreciated as they provide opportunities for company and exchange with other patients in similar situations.

*“I have to stress the advantage of a twin room. If you’re lucky, like I was, you meet a soulmate who you can share your concerns, thoughts and questions with; support in a difficult situation and a new lifelong friendship.” (Patient 56)*

The food was of importance during the hospital stay sometimes as a way of socializing:

*“I felt fine the whole time sat up walked to the dining room ate and socialized with other patients.” (Patient 66)*

### Patients’ experience of health and needs at home after discharge

A total of 60 (75%) patients (*n* = 80) were interviewed by telephone within 2 weeks of discharge. The results are summarized in Table 4 and show that 80% (*n* = 48) felt comfortable coming home with access to their own bed, choosing their own food, and a feeling of freedom with regard to mobilization.

**Table 4:** Telephone interview within 2 weeks of discharge (*n* = 60)

How did you feel coming home?	*n* = 48 felt fine
	*n* = 7 felt coming home was difficult, i.a., becauseof worry/uncertainty about colostomy and being alone
	*n* = 5 felt it was difficult, because of fatigue/pain/nausea
Did you feel that you had received the information you needed to feel safe upon coming home?	*n* = 50 felt well-informed. Written discharge information was very helpful and gave a feeling of safety
	*n* = 9 lacked information on diet/colostomy/surgical dressing
	*n* = 1 received wrong information about list of drugs
How do you feel now, after the operation?	*n* = 24 report fatigue; 11 of these nonetheless report feeling well
	*n* = 20 feel well
	*n* = 7 report fatigue and nausea, pain or worry
	*n* = 4 report pain and/or nausea
	*n* = 4 report worry/depression
	*n* = 1 reports troublesome bowel symptoms
How is your appetite now?	*n* = 28 experience no problems with eating
	*n* = 23 report decreased appetite/eating less
	*n* = 9 report no appetite
If you experience a lack of appetite, what do you think it is due to?	*n* = 8 report nausea
	*n* = 8 report early satiety
	*n* = 4 report food tasting strange/different
	*n* = 4 report not feeling hungry
	*n* = 4 do not know why
	*n* = 4 report other reason
What is the consistency of your stools?	*n* = 15 report loose stools
	*n* = 9 report varying consistency
	*n* = 8 report normal consistency
	*n* = 4 report hard stools/constipation
	*n* = 24 report colostomy
Do you have problems urinating?	*n* = 32 report no problems
	*n* = 11 report problems urinating
	*n* = 9 report urinating more often/frequent need to urinate
	*n* = 3 report incontinence
	*n* = 5 report not being able to discontinue IUC/Carefix*
Do you experience pain around the surgical site? If “yes,” what kind of pain relief do you use?	*n* = 35 report using paracetamol and/or ibuprofen regularly
	*n* = 14 report using no pain relief
	*n* = 8 report taking oxycodone (OxyNorm) as needed
	*n* = 3 report taking paracetamol as needed
Do you have pain anywhere else in your body?	*n* = 17 report already using pain relief because of pain elsewhere
Do you feel strong enough to be up and about now, after the operation? How often? For how long?	*n* = 60 report being up and about and active
	*n* = 36 report resting once or several times per day
	*n* = 21 report walking long distances
How do you feel about me calling you?	*n* = 60 report that the call conveys joy, a feeling of safety and professionalism and that “someone” cares
Do you have any other thoughts and questions?	*n* = 6 have questions about colostomy care and materials
	*n* = 3 have questions about call back/the next appointment—when will that happen?
	*n* = 1 has questions about sick leave
	*n* = 1 has questions about medication

The majority, 85% (*n* = 50), felt well informed in connection with the discharge and the written information they received at the same time was considered helpful.

A commonly reported postoperative symptom was POF, which was experienced by 52% (*n* = 31) of the patients. Nevertheless, many patients, 33% (*n* = 20), described that they felt fairly well and experienced fatigue as natural; something normal after major surgery and not disabling.

Another frequent symptom was affected appetite, where 55% (*n* = 32) experienced poorer appetite or no appetite at all.

A few patients, 13% (*n* = 8), reported normal feces and about half, 47% (*n* = 28), indicated that it was varied, looser, or harder. Patients who received a stoma, 40% (*n* = 24), had difficulty relating to what was “normal” feces.

Almost half (48%, *n* = 28) of the patients had different degrees of difficulty with micturition. In some cases, the patients were still unable to discontinue the urinary catheter.

All patients, 100% (*n* = 60), were active to a greater or lesser extent with one-third, 35% (*n* = 21), walking long distances daily. However, over half of the patients, 60% (*n* = 36) needed to rest once or several times daily.

At home, two-thirds of the patients (68%, *n* = 41) rated their surgical wound pain (NRS) as > 3 and 60% (*n* = 24) of those patients still used postoperative analgesics. One-third of the patients (32%, *n* = 19) rated their pain between 4 and 9, and among these, 17 patients self-medicated regularly with analgesics due to other pain before surgery.

All patients appreciated the contact nurse follow-up call at home postoperatively. It gave a feeling of safety of being remembered. The fact that “someone” cared was perceived as very important.

### Patients’ rated state of health (ERAS-Health) in connection with discharge

Postoperatively (regardless of ASA classification), patients who had undergone laparoscopic surgery rated their state of health more positively in several respects compared with patients who had undergone open surgery (Suppl. Table A). The ASA classification (Suppl. Table B) affects the patients’ state of health postoperatively, especially among women. The surgical method, also affects state of health (Suppl. Table C), were patients classified as ASA I rated their state of health more positively than patients classified as ASA II-III. In an ordinal regression model (Table 2), both the ASA classification and the surgical method seem to affect the patients’ estimated postoperative state of health in several respects. However, it seems that the surgical method affects postoperative state of health to a greater extent than the ASA classification. Sex did not affect the patient’s state of health postoperatively.

## Discussion

This study has given insights into how patients undergoing CR surgery experience their care and state of health within the ERAS program. Patients who had undergone CR surgery within the ERAS program had mainly positive experiences of their hospital stay and most of them felt comfortable coming home. However, experienced state of health is affected by factors like the surgical method, ASA classification, and sex.

In response to the open-ended questions (step 2), the patients expressed “the meaning of information and communication” and described a feeling of safety, which was of great importance for how the patients were able to focus on their rehabilitation postoperatively. This is in agreement with other studies which claim that understandable information (Larsson et al. 2011) makes it easier for patients to handle the pre- and postoperative period as they get an insight into their health condition and treatment, as well as a feeling of increased safety and participation in their self-care (Poland et al. 2017).

However, our results still indicate that there are areas that need to be improved, among them is dietary information. Many patients pointed out that this specific information was inadequate and sometimes even incorrect. According to Wick (Wick, 2013) patients misunderstand, or cannot make use of all the information given by healthcare professionals. In addition, information is mostly generalized and space for “teach-back” is usually not used in a stressed, clinical environment. Health professionals should therefore strive to “read” the patients’ specific needs and act accordingly in order to create trustful agreements throughout the entire perioperative procedure.

The patients also stressed “the meaning of the healthcare professionals’ competence treatment and commitment.” This was described in both positive and negative terms. In this theme, the patients’ perceived pain was often in focus. Several patients described dissatisfaction with how their pain was managed; for example, that the time between pain assessment and pain treatment was experienced as too long and that pain assessment was not performed often enough. In addition, pain relief was sometimes experienced as insufficient. These pain management problems has been highlighted in many different health care contexts over the years (Gunningberg and Idvall, 2007; Guru and Dubinsky, 2000; Phillips et al. 2013).

Within the concept of ERAS, all patients are encouraged to keep a diary to record how much they eat and how often they are active postoperatively. However, research shows that patients lose interest in recording their rehabilitation activities (e.g., eating drinking mobilization) or following instructions related to these activities when health professionals show no interest in the patients’ diary notes (Aasa et al. 2013). Since the patients in our study described the same feeling, an interpretation of our results could be that if the patient’s diary obviously has not been followed up, distrust may develop vis-à-vis the health professional’s commitment. However, we want to emphasize that although some patients experienced distrust and many suffered from POF, the majority were satisfied with the care they received. The patients described satisfaction with speeding up their recovery when they worked with the health professionals in accordance with the guidelines (ERAS).

The patients also emphasized “the meaning of experienced ill-being,” described as a variety of physical and mental experiences. Emotional factors can be related to the patient’s physically perceived pain, as the intensity of pain is affected by distress such as worry, fear, and anxiety. Uncertainty about the life situation generates anxiety and insecurity, which in turn can increase the intensity of the pain (Gorczyca et al. 2013; Martin et al. 2015). The information patients receive preoperatively and how they experience their well-being before surgery will probably be in agreement with how satisfied they are with their care postoperatively. Thorough information before and after surgery is therefore of the utmost importance in order for patients to feel safe, prepared and aware of their recovery, life situation, and potential postoperative symptoms.

Feeling comfortable within the care environment, limited control of privacy, integrity, and lack of community fell into the fourth main theme “the meaning of the healthcare environment and pleasant dining experiences.” Food and meals are important to most people and, in addition to providing the necessary energy, a good taste experience can promote a sense of social affinity and well-being. In our study, the importance of food seemed to be a factor that contributed to the patients’ well-being. Different degrees of concern were also experienced. “Just” being cared for in a room with a stranger can be perceived as unpleasant and as being exposed. On the other hand, patients who felt fellowship with their “roommates” or received visits from supportive relatives experienced a sense of safety with someone being close to them, someone they could share their situation with*.* This result is confirmed by other studies showing that patients appreciated being cared for in rooms with multiple beds as it created opportunities to socialize with people in similar situations and not feeling alone. Sharing suffering, supporting each other, sharing life perspectives, and situations could lead to increased well-being (Hoybye, 2013; Rowlands and Noble, 2008). The disadvantages were having to disturb their “roommates” at night through frequent toilet visits, while sharing a room also affected one’s own integrity and privacy negatively.

The patients appreciated coming home, with access to their own things and private life, although many patients had problems with their appetite, POF, and micturition. A commonly described symptom was POF. It has been shown that nearly three-quarters of patients undergoing major abdominal surgery experience POF up to 3 months postoperatively, thus much longer than, for example, postoperative pain (DeCherney et al. 2002; Jakobsson et al. 2014). Despite POF, the patients in our study reported that they were doing well and described this symptom as natural and normal after major surgery and that it was not perceived as disabling; hence, all patients were active to a greater or lesser extent. Within the ERAS concept, patients are encouraged to increase their physical activity before surgery to mobilize early after surgery and to engage in daily physical activity after discharge. In cancer-related fatigue, physical activity can be an important part of symptom relief (Kangas et al. 2008). This implies that physical activity has a symptom-reducing effect, which probably contributes to faster recovery and better HRQoL. Postoperatively, many patients also described problems with the appetite feeling nauseated as well as concerned about eating the wrong food, which would make them constipated. This problem has been reported earlier, however, with different frequencies and percentages (Burch and Taylor, 2012; Norlyk and Harder, 2011; Wennström et al. 2010).

Postoperative pain at home was not a symptom that was markedly present in our study. This is in line with a review including ten studies with CR surgery patients within the ERAS program, concluding that patients have relatively little postoperative pain after discharge (Khan et al. 2010). Noteworthy, in our study is that many patients already used analgesics for other reasons, which could affect the results.

Almost all patients reported that they felt safe and well informed in connection with their discharge. The written information was especially appreciated, since this assured them that they would find answers to questions that arise “here and now.” However, some patients felt insecure due to both insufficient information during the hospital stay and lack of time when they received information in connection with discharge. This is in agreement with other studies, which claim that insufficient information to the patient, affects both the content of the information and the patients’ ability to ask questions. This in turn creates a feeling of uncertainty and of not being seen (Aasa et al. 2013; Walker, 2007).

All patients appreciated being called at home by the contact nurse. It gave them a feeling of safety of being remembered and of someone caring. In line with Jonsson et al. (Jonsson et al. 2011), our study indicates that the contact nurse follow-up call fulfills an important role for the patients. Not only because they get the opportunity to ask questions about what worries them but also for the encouragement they get to find normality and guidance for their continued rehabilitation. The contact nurse can supply additional information that the patient has not picked up during the hospital stay or in connection with the discharge. This is in agreement with other study results that strongly emphasize the need for improvement particularly with regard to post-discharge support from both community services and hospital follow-up (Bernard and Foss, 2014; Burch and Taylor, 2012).

A meta-analysis shows faster recovery as well as reduced complications and hospital stay if surgery is performed laparoscopically (Lu et al. 2019). Studies also show that both laparoscopic surgery and care using the ERAS program could prevent POF (Rorarius et al. 2001; Schwenk et al. 1998; Zargar-Shoshtari et al. 2009).

A randomized Danish study (Basse et al. 2005) comparing patients undergoing laparoscopic and open CR surgery with “fast-track rehabilitation,” showed no differences postoperatively with regard to the length of hospital care, pain, nausea, fatigue, or sleep quality. Unlike Basse et al., the present study (Table 2, Suppl. Table A and C) shows that patients operated on with open surgery rated their state of health (including the variables mentioned above) less positively in many respects than patients undergoing laparoscopic surgery. One explanation for this might be that the patients in our study were cared within a “modern” (year 2016-2018) ERAS program which is evolving continuously, based on the best available evidence (Gustafsson et al. 2019).

### Methodological considerations

To create variations in perspectives regarding the patients’ experience of health and care, both a quantitative (ERAS-HEALTH questionnaire) and a qualitative method (open-ended questions) as well as quantitative content analysis (telephone interviews) were used. There is always a risk of a large number of dropouts when a questionnaire is used (Wang, 2015). In this study, the patients—often depending on their degree of tiredness—could choose to fill in the questionnaire at the hospital in connection with discharge, or after a few days at home. The ERAS-HEALTH response rate was 100%, and 81% responded to the open-ended questions.

The ERAS-HEALTH has never been validated in its entirety. However, in its design the ERAS-HEALTH is similar to the ESAS (Bruera et al. 1991) with the exception of the addition of the two open-ended questions.

The researcher’s interaction is described in terms of reflexivity and relationality, the former referring to the researcher being a part of rather than being separated from the data, while the latter addresses power and trust in the relationship between the participant and the researcher (Hall and Callery, 2001). Improving rigour around these issues also includes the idea that the researcher identifies and reflects on the preconceptions he or she brings into the study. This will not be the same as bias unless the researcher fails to acknowledge them (Malterud, 2001). The analysis of the data may have been influenced by the fact that the preconceptions of three of the authors (BW, AJ, SK) are based on a nursing science perspective, encompassing knowledge, experience and a sense of duty and commitment accumulated over many years and caring for this kind of patients at different stages of their illness, both before around and after their operation: a professional pre-understanding. This knowledge may be a strength in the project but may also be a limitation. On the other hand, these previous insights may have increased the trustworthiness of the study as knowledge about the diagnosis, illness, treatment, and care, facilitated the condensation and analysis of the data.

During the course of the process, the authors ensured that the analysis corresponded to the aim and that all data were analyzed at the same time, separately by each author, and by the authors together. The authors’ different experiences made it possible to challenge each other’s pre-understanding and to return continuously to the data for confirmation of interpretations as well as reflection on methodological procedures. This can be seen as a strength with regard to ensuring trustworthiness. The context and the participants are also described as clearly as possible to facilitate the transferability of the results.

During the summer vacation period (June-July), there are low staffing levels and, hence, often a heavy workload. Since there was not enough time for the nurses to inform and include the patients properly during this period, patients were not invited to participate in the study. However, all patients in our institution follow the guidelines of the ERAS program strictly.

## Conclusions

The patients reported many positive aspects as well as challenges when being cared for within the ERAS program. They favored the program particularly due to the early discharge, as home was the preferred place for recovery. However, due to lack of information they experienced some insecurity most prominent at home, postoperatively.

Our study emphasizes the need to give the patients not only standardized information but also *personalized* information. This is intended to ensure self-recovery and well-being and should provide health professionals with knowledge of how to support ERAS patients, both pre- and postoperatively.

- POF is a common problem for patients after CR surgery. Other problems are related to insufficient information, food/food intake, appetite, pain management, micturition ostomy care, and the care environment.
- Despite several problems postoperatively, most patients reported positive experiences regarding their hospital care and most of them felt comfortable coming home.
- The postoperative call at home is important and probably as important as the pre-and postoperative information that the patient receives at the hospital.
- Laparoscopic surgery affects the patients’ state of health positively in several respects compared with open surgery.

### Clinical implications and further research

It is important to involve the patients in their own rehabilitation and to give them the ability to control their situation. Therefore, preparation for support and information is required including information about setbacks that may occur postoperatively (for example at home). This could be made possible through an information booklet created *together* with the patient, describing which problems they may come across and what resources the patient needs to handle regarding different situations that may arise postoperatively. It can also provide coherence to the information provided by the health professionals (nurses, physicians, nurse assistants, dieticians/physio-therapists/occupational, therapists etc.). This concept includes routine postoperative telephone calls at home as they fulfill an important function for the patient’s safety and continued rehabilitation in relation to well-being and HRQoL. This study is not consecutive—although we believe it includes a representative sample of colorectal ERAS patients—but presents results that make it possible for us to improve the ERAS concept further.

## Supplementary information

**Additional file 1:** Table A. Differences between surgical methods with respect to experienced state of health among men and women separately. Table B. Differences between ASA classifications with respect to experienced state of health among men and women separately. Table C. Differences between surgical methods with respect to experienced state of health among ASA classification I and ASA classification II-III separately.

## Data Availability

According to the Swedish law—The Patient Data Act—data from this research will not be shared to ensure data confidentiality.

## Abbreviations

ASA: American Society of Anesthesiologists (physical status classification)
CS: Colorectal surgery
ERAS: Enhanced recovery after surgery
ESAS: Edmonton symptom assessment scale
HRQoL: Health-related quality of life
NRS: Numerical rating scale
POF: Postoperative fatigue
PONV: Postoperative nausea and vomiting
