# Supplementary material for: Patient experience of health and care when undergoing colorectal surgery within the ERAS program
Source: Perioper Med (Lond). 2020 May 20;9:15. doi: 10.1186/s13741-020-00144-6 (PMC7238535; doi:10.1186/s13741-020-00144-6)
Supplement: Supplementary file 1 — Additional file 1: Table A. Differences between surgical methods with respect to experienced state of health among men and women separately. Table B. Differences between ASA classifications with respect to experienced state of health among men and women separately. Table C. Differences between surgical methods with respect to experienced state of health among ASA classification I and ASA classification II-III separately. [file 13741_2020_144_MOESM1_ESM.docx]

| **Table A. Differences between surgical methods with respect to experienced state of health among men and women separately.** | | | | | | |
| --- | --- | --- | --- | --- | --- | --- |
|  | **Laparoscopic surgery** | | **Open surgery** | |  |  |
| **State of Health** | **Mean (SD)** | **Median (min, max)** | **Mean (SD)** | **Median (min, max)** | **p-value** |  |
| **Pain**  Men  Women | 3.54 (1.75)  3.58 (1.54)  3.50 (1.95) | 3.0 (1-8)  3.0 (1-7)  3.0 (1-8) | 4.21 (2.52)  3.92 (2.43)  4.71 (2.67) | 3.5 (1-10)  3.0 (1-9)  4.5 (1-10) | 0.379  0.990  0.162 |  |
| **Worry/anxiety**  Men  Women | 2.76 (1.94)  2.95 (1.96)  2.61 (1.95) | 2.0 (1-8)  2.0 (1-8)  2.0 (1-7) | 2.92 (2.39)  2.79 (2.34)  3.14 (2.54) | 2.0 (1-8)  2.0 (1-8)  2.0 (1-8) | 0.846  0.449  0.615 |  |
| **Fatigue**  Men  Women | 3.07 (1.75)  2.95 (1.84)  3.18 (1.71) | 3.0 (1-8)  3.0 (1-8)  3.0 (1-7) | 4.47 (2.18)  4.13 (2.01)  5.07 (2.40) | 4.0 (1-8)  4.0 (1-8)  5.5 (1-8) | **0.005***  0.057  **0.017*** |  |
| **Nausea**  Men  Women | 1.64 (1.10)  1.42 (0.77)  1.83 (1.30) | 1.0 (1-5)  1.0 (1-3)  1.0 (1-5) | 3.03 (2.78)  2.67 (2.55)  3.64 (3.13) | 2.0 (1-10)  1.0 (1-10)  2.0 (1-10) | **0.025***  0.107  0.060 |  |
| **Depression**  Men  Women | 2.10 (1.38)  2.21 (1.55)  2.00 (1.24) | 1.5 (1-5)  1.0 (1-5)  2.0 (1-5) | 2.61 (1.98)  2.04 (1.30)  3.57 (2.56) | 2.0 (1-8)  2.0 (1-5)  3.0 (1-8) | 0.306  0.937  0.060 |  |
| **Drowsiness**  Men  Women | 2.93 (1.72)  2.58 (1.64)  3.22 (1.76) | 3.0 (1-7)  3.0 (1-7)  3.0 (1-7) | 4.11 (2.01)  3.92 (1.79)  4.43 (2.38) | 4.0 (1-8)  4.0 (1-8)  4.5 (1-8) | **0.008***  **0.013***  0.135 |  |
| **Appetite**  Men  Women | 3.95 (2.14)  4.05 (1.93)  3.87 (2.34) | 3.0 (1-8)  3.0 (1-7)  3.0 (1-8) | 5.26 (2.73)  5.25 (2.97)  5.29 (2.37) | 5.0 (1-10)  4.5 (1-10)  5.5 (1-8) | **0.026***  0.164  0.082 |  |
| **Breathlessness**  Men  Women | 1.88 (1.38)  2.16 (1.43)  1.64 (1.33) | 1.0 (1-7)  2.0 (1-7)  1.0 (1-7) | 2.55 (2.01)  2.67 (2.18)  2.36 (1.74) | 2.0 (1-9)  2.0 (1-9)  1.0 (1-5) | 0.229  0.798  0.317 |  |
| **Well-being**  Men  Women | 2.86 (1.84)  3.05 (1.84)  2.70 (1.87) | 2.5 (1-7)  3.0 (1-7)  2.0 (1-7) | 4.39 (2.48)  4.17 (2.35)  4.79 (2.72) | 4.5 (1-10)  4.0 (1-10)  5.0 (1-9) | **0.004***  0.118  **0.018*** |  |
| **Quality of Life**  Men  Women | 2.76 (1.95)  2.84 (1.64)  2.70 (2.20) | 2.0 (1-10)  3.0 (1-6)  2.0 (1-10) | 4.58 (2.52)  4.21 (2.38)  5.21 (2.72) | 5.0 (1-10)  4.5 (1-10)  5.0 (1-9) | **0.001***  0.056  **0.007*** |  |

Significant differences, p<0.05, in bold (Mann-Whitney U test).

| **Table B. Differences between ASA classifications with respect to experienced state of health among men and women separately.** | | | | | |  |
| --- | --- | --- | --- | --- | --- | --- |
|  | **ASA classification I** | | **ASA classification II-III** | |  | |
| **State of Health** | **Mean (SD)** | **Median (min-max)** | **Mean (SD)** | **Median (min-max)** | **p-value** | |
| **Pain**  Men  Women | 3.04 (1.67)  3.60 (1.71)  2.62 (1.56) | 3.0 (1-8)  3.0 (2-8)  2.0 (1-6) | 4.20 (2.27)  3.82 (2.19)  4.74 (2.32) | 4.0 (1-10)  3.0 (1-9)  5.0 (1-10) | **0.030***  0.895  **0.005*** | |
| **Worry/anxiety**  Men  Women | 3.13 (2.47)  4.00 (2.75)  2.46 (2.11) | 2.0 (1-8)  3.5 (1-8)  2.0 (1-7) | 2.72 (2.02)  2.52 (1.86)  3.00 (2.23) | 2.0 (1-8)  2.0 (1-8)  2.5 (1-8) | 0.613  0.112  0.449 | |
| **Fatigue**  Men  Women | 2.77 (1.66)  3.40 (2.01)  2.25 (1.14) | 2.0 (1-8)  3.0 (1-8)  2.0 (1-4) | 4.12 (2.11)  3.67 (2.03)  4.75 (2.11) | 4.0 (1-8)  3.0 (1-8)  5.0 (1-8) | **0.008***  0.693  **0.001*** | |
| **Nausea**  Men  Women | 2.17 (2.50)  2.80 (2.82)  1.69 (2.21) | 1.0 (1-10)  2.0 (1-10)  1.0 (1-9) | 2.35 (2.05)  1.91 (1.76)  2.96 (2.29) | 1.0 (1-10)  1.0 (1-8)  2.0 (1-10) | 0.353  0.153  **0.007*** | |
| **Depression** Men  Women | 2.17 (1.61)  2.40 (1.43)  2.00 (1.78) | 1.0 (1-7)  2.5 (1-5)  1.0 (1-7) | 2.40 (1.74)  2.03 (1.40)  2.92 (2.04) | 2.0 (1-8)  1.0 (1-5)  2.0 (1-8) | 0.519  0.406  0.076 | |
| **Drowsiness**  Men  Women | 2.61 (1.75)  3.00 (2.11)  2.31 (1.44) | 2.0 (1-7)  2.0 (1-7)  2.0 (1-5) | 3.84 (1.92)  3.42 (1.77)  4.42 (2.00) | 4.0 (1-8)  3.0 (1-10)  4.5 (1-8) | **0.009***  0.429  **0.003*** | |
| **Appetite**  Men  Women | 4.52 (2.59)  5.30 (2.79)  3.92 (2.36) | 5.0 (1-10)  4.5 (2-10)  5.0 (1-8) | 4.60 (2.50)  4.55 (2.56)  4.67 (2.46) | 4.0 (1-10)  4.0 (1-10)  4.5 (1-8) | 0.872  0.434  0.336 | |
| **Breathlessness**  Men  Women | 2.22 (1.70)  3.00 (2.00)  1.62 (1.19) | 1.0 (1-6)  2.5 (1-6)  1.0 (1-5) | 2.20 (1.76)  2.27 (1.84)  2.09 (1.68) | 2.0 (1-9)  2.0 (1-9)  1.0 (1-7) | 0.931  0.265  0.408 | |
| **Well-being**  Men  Women | 3.57 (2.23)  4.00 (2.26)  3.23 (2.24) | 3.0 (1-8)  4.5 (1-8)  3.0 (1-8) | 3.60 (2.33)  3.58 (2.19)  3.63 (2.55) | 3.0 (1-10)  3.0 (1-10)  3.0 (1-9) | 0.978  0.503  0.783 | |
| **Quality of Life**  Men  Women | 3.83 (2.42)  4.20 (1.99)  3.54 (2.76) | 3.0 (1-10)  4.0 (1-8)  3.0 (1-10) | 3.54 (2.41)  3.42 (2.22)  3.71 (2.69) | 3.0 (1-10)  3.0 (1-10)  3.0 (1-9) | 0.567  0.231  0.935 | |

Significant differences, p<0.05, in bold (Mann-Whitney U test).

| **Table C. Differences between surgical methods with respect to experienced state of health among ASA classification I and ASA classification II-III separately.** | | | | | |
| --- | --- | --- | --- | --- | --- |
|  | **Laparoscopic surgery** | | **Open surgery** | |  |
| **State of health** | **Mean (SD)** | **Median (min-max)** | **Mean (SD)** | **Median (min-max)** | **p-value** |
| **Pain**  ASA I  ASA II-III | 3.54 (1.75)  2.71 (1.14)  3.96 (1.87) | 3.0 (1-8)  3.0 (1-5)  4.0 (1-8) | 4.21 (2.52)  3.56 (2.24)  4.41 (2.60) | 3.5 (1-10)  3.0 (1-8)  4.0 (1-10) | 0.497  0.734 |
| **Worry/anxiety**  ASA I  ASA II-III | 2.76 (1.94)  3.00 (2.57)  2.64 (1.57) | 2.0 (1-8)  2.0 (1-8)  2.5 (1-6) | 2.92 (2.39)  3.33 (2.45)  2.79 (2.40) | 2.0 (1-8)  2.0 (1-8)  2.0 (1-8) | 0.535  0.596 |
| **Fatigue**  ASA I  ASAII-III | 3.07 (1.75)  2.38 (1.21)  3.39 (1.91) | 3.0 (1-8)  2.0 (1-5)  4.0 (1-8) | 4.47 (2.18)  3.33 (2.18)  4.83 (2.09) | 4.0 (1-8)  4.0 (1-8)  5.0 (1-8) | 0.346  **0.016*** |
| **Nausea**  ASA I  ASA II-III | 1.64 (1.10)  1.21 (0.43)  1.86 (1.27) | 1.0 (1-5)  1.0 (1-2)  1.0 (1-5) | 3.03 (2.78)  3.67 (3.57)  2.83 (2.52) | 2.0 (1-10)  2.0 (1-10)  2.0 (1-10) | **0.041***  0.202 |
| **Depression**  ASA I  ASA II-III | 2.10 (1.38)  1.64 (1.01)  2.32 (1.49) | 1.5 (1-5)  1.0 (1-4)  2.0 (1-5) | 2.61 (1.98)  3.00 (2.06)  2.48 (1.98) | 2.0 (1-8)  3.0 (1-7)  2.0 (1-8) | 0.076  0.993 |
| **Drowsiness**  ASA I  ASAII-III | 2.93 (1.72)  2.07 (1.33)  3.36 (1.75) | 3.0 (1-7)  2.0 (1-5)  3.0 (1-7) | 4.11 (2.01)  3.44 (2.07)  4.31 (1.98) | 4.0 (1-8)  4.0 (1-7)  4.0 (1-8) | 0.101  0.065 |
| **Appetite**  ASA I  ASA II-III | 3.95 (2.14)  3.57 (2.07)  4.14 (2.19) | 3.0 (1-8)  3.0 (1-7)  3.5 (1-8) | 5.26 (2.73)  6.00 (2.74)  5.03 (2.73) | 5.0 (1-10)  5.0 (1-10)  4.0 (1-10) | **0.049***  0.206 |
| **Breathlessness**  ASA I  ASA II-III | 1.88 (1.38)  1.57 (0.76)  2.04 (1,61) | 1.0 (1-7)  1.0 (1-3)  2.0 (1-7) | 2.55 (2.01)  3.22 (2.28)  2.34 (1.91) | 2.0 (1-9  3.0 (1-6)  2.0 (1-9) | 0.133  0.642 |
| **Well-being**  ASA I  ASA II-III | 2.86 (1.84)  3.00 (1.80)  2.79 (1.89) | 2.5 (1-7)  3.0 (1-7)  2.0 (1-7) | 4.39 (2.48)  4.44 (2.65)  4.38 (2.47) | 4.5 (1-10)  5.0 (1-8)  4.0 (1-10) | 0.191  **0.013*** |
| **Quality of Life**  ASA I  ASA II-III | 2.76 (1.95)  3.21 (2.29)  2.54 (1.75) | 2.0 (1-10)  3.0 (1-10)  2.0 (1-6) | 4.58 (2.52)  4.78 (2.44)  4.52 (2.59) | 5.0 (1-10)  5.0 (1-8)  5.0 (1-10) | 0.090  **0.002*** |

Significant differences, p<0.05, in bold (Mann-Whitney U test).
